# Association of biological aging with prostate cancer: insights from the National Health and Nutrition Examination Survey

**DOI:** 10.1007/s40520-024-02861-0

**Published:** 2024-10-24

**Authors:** Weiqi Yin, Baiyang Song, Chengling Yu, Junhui Jiang, Zejun Yan, Chengxin Xie

**Affiliations:** 1 Department of Urology, The First Affiliated Hospital of Ningbo University, Ningbo, Zhejiang China; 2 Ningbo Clinical Research Center for Urological Disease, Ningbo, Zhejiang China; 3 Zhejiang Engineering Research Center of Innovative Technologies and Diagnostic and Therapeutic Equipment for Urinary System Diseases, Ningbo, Zhejiang China; 4 Department of Orthopedics, Taizhou Hospital of Zhejiang Province Affiliated to Wenzhou Medical University, Taizhou, Zhejiang China

**Keywords:** Biological aging, KDMAge, PhenoAge, Prostate cancer, Prostate specific antigen, National Health and Nutrition Examination Survey

## Abstract

The link between biological aging and prostate cancer (PCa) risk, particularly as indicated by elevated prostate-specific antigen (PSA) levels, remains uncertain. This study utilized data from the National Health and Nutrition Examination Survey (2001–2010) to explore this association. Biological age was assessed using Klemera-Doubal method age (KDMAge) and phenotypic age (PhenoAge). PCa was identified through self-reported diagnoses, and highly probable PCa was determined by PSA levels. We analyzed the prevalence of PCa and PSA-defined highly probable PCa across quartiles of biological age measures using weighted chi-square and linear trend tests. Associations were evaluated using weighted multiple logistic regression models. Among 7,209 and 6,682 males analyzed, the overall weighted prevalence of PCa was 2.86%, increasing to 9.60% in those aged 65 and above. A significant rise in PCa prevalence was observed with higher quartiles of KDMAge or PhenoAge (P for trend < 0.001), particularly in those under 65. In this younger group, higher PhenoAge acceleration quartiles were linked to increased PCa prevalence and higher risk of PCa (OR = 1.50, P = 0.015) as well as highly probable PCa in those without a diagnosis (OR = 1.28, P = 0.031). These findings suggest that accelerated biological aging is associated with an increased risk of PCa and may indicate early risk as signaled by PSA levels, even in those without a PCa diagnosis.

**Supplementary Information:**

The online version contains supplementary material available at 10.1007/s40520-024-02861-0.

## Introduction

Prostate cancer (PCa) has become a rising cause of mortality, accounting for 15% of all male cancers. Global incidence of PCa is projected to rise from 1.4 million annual cases in 2020 to 2.9 million by 2040, with annual deaths anticipated to increase from 375,000 to nearly 700,000, an increase of about 85% [1]. This surge is expected to impose a significant burden on individuals and societies alike. Consequently, a refined classification scheme is urgently required to facilitate early and accurate identification of high-risk PCa, which would aid in developing more effective treatment paradigms.

Aging is a high-risk factor for PCa, with the incidence of the disease escalating with age. The mean age of affected patients ranges from 72 to 74 years, with about 85% diagnosed post 65 years [2]. A European study indicated that the prevalence of incidental PCa in males aged 30–69 was 30%, which rose to 75% in those over 70 years old [3]. However, a study assessing the trends in metastatic PCa in the U.S. population by age and race revealed that the annual growth rate of metastatic PCa in men under 69 years old was faster than in older men, with the highest projected growth rates in males aged 45–54 years old [4]. While chronological age plays a crucial role in predicting prostate cancer risk, its limitations in representing the true physiological and pathological disorders underlying aging cannot be ignored, possibly explaining the increasing risk among younger individuals. Hence, identifying indicators that more accurately reflect true human aging and exploring their association with PCa risk could better identify the residual risk independent of chronological age.

Biological aging, which refers to the aging processes in multiple biological systems [5], is a leading risk factor for most age-related diseases, physical and cognitive impairments, and mortality [6]. Increasing studies have linked biological aging closely with cancer [7–9]. Hallmarks of aging, such as genome instability, cellular senescence, and epigenetic changes, also overlap with the hallmarks of cancer [9]. Recent research has corroborated the association between aging and PCa in humans. However, biological aging captures the heterogeneity among older individuals [10]. Biological age, on the other hand, integrates information from biological markers and may better reflect an individual's physiological state and risks of age-related diseases and death [10]. Biological age calculated using the KDMAge and PhenoAge, based on a set of easily obtainable clinical measurements and blood test markers, provides a comprehensive assessment of a person's biological age [11]. Compared to chronological age, identifying the biological age of individuals can facilitate timely interventions to prevent the onset of diseases [12]. However, it remains to be determined whether measurements of biological age can serve as a surrogate for biological aging in predicting the incidence of PCa.

In this study, we explore the impact of biological age on PCa in a nationally representative population using cross-sectional data from the National Health and Nutrition Examination Surveys (NHANES). We also analyze the relationship between biological age acceleration and elevated levels of prostate-specific antigen (PSA), indicating a high risk in individuals without PCa. This research aims to provide new insights for early identification and intervention of aging-related prostate cancer risks in clinical settings.

## Methods

### Study design and population

Data for this study were derived from the NHANES, a cross-sectional survey designed to assess the health and nutritional status of the American population [13]. NHANES encompasses demographic, socioeconomic, physical examinations, biospecimen collection, and questionnaire data. Our analysis utilized combined data from five cycles spanning 2001–2010. After excluding individuals under 40 years old, females, and those with missing key indicators, a total of 7,209 male participants were included for analysis related to biological age and PCa. Due to the absence of PSA testing in part individuals with a history of PCa and those who had undergone recent procedures affecting PSA levels, 6,682 participants were included for analyzing the correlation between biological age and PSA-involved metrics. Fig.S1 illustrated the detailed participant selection process. Ethical approval for the study was granted by the NCHS Ethics Review Board, and all NHANES participants provided written informed consent. Detailed information about the NHANES database is available at https://www.cdc.gov/nchs/nhanes/index.htm.

### Biological aging assessment

Biological aging was quantified using two biological age measurement methods: the Klemera-Doubal method age (KDMAge) and phenotypic age (PhenoAge). KDMAge predicts an individual's age under typical physiological conditions and is calculated via regression based on clinical indicators including systolic blood pressure, albumin, alkaline phosphatase, blood urea nitrogen, creatinine, C-reactive protein, glycated hemoglobin, and total cholesterol concentrations. PhenoAge is derived from a multivariate analysis of mortality risk, calculated using clinical markers including albumin, alkaline phosphatase, creatinine, glucose, C-reactive protein concentrations, lymphocyte percentage, mean cell volume, red cell distribution width, and white blood cell count. Biological age residuals, calculated by regressing biological age measures against the age at which biomarkers were measured, were defined as KDMAge acceleration and PhenoAge acceleration to evaluate accelerated biological aging. Acceleration values greater than zero were classified as accelerated KDMAge or PhenoAge, and values less than or equal to zero were classified as non-accelerated KDMAge or PhenoAge.

### Assessment of PCa diagnosis and highly-probable PCa

PCa was identified based on self-reported previous diagnoses from questionnaire item KIQ201 in the PSA detection section of the Laboratory Data. PSA testing was applicable to male participants aged 40 and older, excluding those with recent prostatic interventions or inflammation, or a history of PCa. Serum free PSA and total PSA concentrations were measured using immunoassays (Hybritech tests, Beckman Coulter, Fullerton, CA). The PSA ratio was calculated by dividing free PSA by total PSA. Individuals with total PSA levels exceeding 10 ng/mL or between 4–10 ng/mL with a PSA ratio less than 10% were considered highly-probable for PCa.

### Covariates

A comprehensive set of covariates was considered to potentially confound the relationship between biological age measures and PCa risk, including chronological age, body mass index (BMI), race, obesity, history of diabetes, history of hypertension, smoking status, and alcohol consumption. Races included "Mexican American," "Other Hispanic," "Non-Hispanic White," "Non-Hispanic Black," and "Other Race—Including Multi-Racial." Obesity was defined as a BMI over 30 kg/m^2^. Histories of diabetes and hypertension were identified based on self-reported diagnoses. Smoking status was categorized as "Not at all," "Some days," or "Every day." Alcohol consumption was categorized based on whether individuals consumed at least 12 alcoholic drinks in the previous year. Due to the strong correlation between PCa and aging, the population was further divided into younger (< 65 years) and older (≥ 65 years) groups for subsequent subgroup analyses [2].

### Statistical analysis

Due to NHANES’ complex multistage probability sampling design, five two-year cycle weights were calculated and applied in all analyses to provide nationally representative estimates. Continuous variables were presented as medians with interquartile ranges and compared using the weighted Wilcoxon rank-sum test, while categorical variables were described by weighted percentages and compared using the weighted chi-squared test. Weighted linear regression models examined the linear trends for PCa prevalence or percentage of highly-probable PCa with increasing quartiles of biological age measures. After adjusting for covariates, the associations between biological age measures and the risk of PCa or highly-probable PCa were assessed using weighted multiple logistic regression models, presenting results as odds ratios (ORs) with 95% confidence intervals (CIs) and p-values. All biological age measures were standardized. Due to the strong correlation between PCa and aging, the population was further divided into younger (< 65 years) and older (≥ 65 years) groups for subsequent subgroup analyses. A two-sided p-value of less than 0.05 was considered statistically significant. All statistical analyses were conducted using R software version 4.3.1.

## Results

### Basic characteristics of the study participants

A total of 7,209 participants analyzed for PCa were stratified by age into younger (< 65 years) and older (≥ 65 years) groups, with their unweighted and weighted demographic details presented in Table 1. The younger group had a weighted median age of 50 years, representing approximately 41,308,362 individuals nationwide, while the older group had a weighted median age of 72 years, representing approximately 12,759,720 individuals. The younger group exhibited higher BMI and prevalence of obesity, as well as higher frequencies of smoking and alcohol consumption. The older group had higher proportions of individuals with histories of diabetes and hypertension. For individual biological age measures, the older group showed higher median levels of systolic blood pressure, glucose, glycated hemoglobin, creatinine, blood urea nitrogen, C-reactive protein, mean cell volume, and red cell distribution width, and lower median levels of total cholesterol, albumin, and lymphocyte percentage. There were no significant differences in alkaline phosphatase and white blood cell count between the groups. As expected, the older group displayed higher levels of KDMAge and PhenoAge. However, compared to the younger group, the older group had lower levels of KDMAge acceleration and a lower proportion of accelerated KDMAge, which differed from the performance of PhenoAge acceleration, with the older group having higher levels of PhenoAge acceleration and a higher proportion of accelerated PhenoAge. The overall weighted prevalence of PCa was 2.86%, with the younger group showing a weighted prevalence of 0.78% and the older group as high as 9.60%. The weighted median levels of total PSA and free PSA were higher in the older group compared to the younger group, while levels of PSA ratios between the two age groups showed no significant differences.

**Table 1:** Basic characteristics of study population

	Unweighted				Weighted			
	Overall	Age < 65 years	Age ≥ 65 years	P value	Overall	Age < 65 years	Age ≥ 65 years	P value
N	7209	4520	2689		54,068,082	41,308,362	12,759,720	
Age (years)	60 (49 ~ 70)	51 (45 ~ 58)	74 (69 ~ 80)	< 0.001	53 (46 ~ 64)	50 (45 ~ 55)	72 (68 ~ 78)	< 0.001
Race (n %)				< 0.001				< 0.001
Mexican American	1248 (17.31)	896 (19.82)	352 (13.09)		3,132,781 (5.79)	2,675,075 (6.48)	457,706 (3.59)	
Other Hispanic	425 (5.90)	311 (6.88)	114 (4.24)		1,828,077 (3.38)	1,509,191 (3.65)	318,886 (2.50)	
Non-Hispanic	4003 (55.53)	2232 (49.38)	1771 (65.86)		42,171,414 (78)	31,405,894 (76.03)	10,765,520 (84.37)	
Non-Hispanic	1304 (18.09)	925 (20.46)	379 (14.09)		4,678,547 (8.65)	3,853,620 (9.33)	824,927 (6.47)	
Other Race	229 (3.18)	156 (3.45)	73 (2.71)		2,257,262 (4.17)	1,864,582 (4.51)	392,680 (3.08)	
BMI (kg/m^2^)	28.03 (25.09 ~ 31.34)	28.32 (25.41 ~ 31.83)	27.52 (24.69 ~ 30.65)	< 0.001	28.21 (25.36 ~ 31.54)	28.34 (25.50 ~ 31.72)	27.81 (24.86 ~ 31.03)	< 0.001
Obesity (n %)	2404 (33.35)	1634 (36.15)	770 (28.64)	< 0.001	18,766,376 (34.71)	14,851,031 (35.95)	3,915,345 (30.69)	0.001
**ALT (U/L)**	24 (19 ~ 32)	26 (21 ~ 35)	20 (17 ~ 26)	< 0.001	25 (20 ~ 33)	27 (21 ~ 35)	21 (17 ~ 27)	< 0.001
**AST (U/L)**	25 (21 ~ 30)	25 (21 ~ 31)	24 (21 ~ 28)	< 0.001	25 (21 ~ 30)	25 (21 ~ 30)	24 (21 ~ 28)	< 0.001
History of diabetes (n %)	1049 (14.55)	538 (11.90)	511 (19.00)	< 0.001	5,821,403 (10.77)	3,574,886 (8.65)	2,246,516 (17.61)	< 0.001
History of hypertension (n %)	3040 (42.17)	1557 (34.45)	1483 (55.15)	< 0.001	20,501,817 (37.92)	13,438,275 (32.53)	7,063,541 (55.36)	< 0.001
Smoking status (n %)				< 0.001				< 0.001
Never	5598 (77.65)	3223 (71.31)	2375 (88.32)		42,310,437 (78.25)	30,768,445 (74.48)	11,541,995 (90.46)	
Sometimes	233 (3.23)	200 (4.42)	33 (1.23)		1,339,889 (2.48)	1,252,927 (3.03)	86,962 (0.68)	
Everyday	1378 (19.11)	1097 (24.27)	281 (10.45)		10,417,756 (19.27)	9,286,992 (22.48)	1,130,764 (8.86)	
Drinking (n %)	5668 (78.62)	3646 (80.66)	2022 (75.20)	< 0.001	43,427,783 (80.32)	33,770,368 (81.75)	9,657,415 (75.69)	< 0.001
Components included in BA algorithms								
Systolic blood pressure (mmHg)	126 (116 ~ 139)	123.33 (115 ~ 135)	132 (120 ~ 146)	< 0.001	124 (115 ~ 135)	122.67 (115 ~ 133)	130 (119 ~ 143)	< 0.001
Total cholesterol (mg/dL)	196 (170 ~ 225)	203 (177 ~ 231)	184 (159 ~ 213)	< 0.001	199 (173 ~ 227)	203 (178 ~ 231)	184 (158 ~ 212)	< 0.001
Glucose (mmol/L)	5.33 (4.88 ~ 6.05)	5.27 (4.83 ~ 5.83)	5.50 (5.00 ~ 6.38)	< 0.001	5.27 (4.83 ~ 5.83)	5.22 (4.83 ~ 5.72)	5.50 (5.00 ~ 6.33)	< 0.001
Glycated hemoglobin (%)	5.60 (5.30 ~ 6.00)	5.50 (5.30 ~ 5.90)	5.70 (5.40 ~ 6.10)	< 0.001	5.50 (5.30 ~ 5.80)	5.50 (5.20 ~ 5.70)	5.70 (5.40 ~ 6.00)	< 0.001
Albumin (g/dL)	4.30 (4.10 ~ 4.40)	4.30 (4.10 ~ 4.50)	4.20 (4 ~ 4.40)	< 0.001	4.30 (4.10 ~ 4.50)	4.30 (4.20 ~ 4.50)	4.20 (4 ~ 4.40)	< 0.001
Creatinine (mg/dL)	1.00 (0.90 ~ 1.11)	0.98 (0.87 ~ 1.10)	1.05 (0.91 ~ 1.20)	< 0.001	1.00 (0.90 ~ 1.10)	1.00 (0.90 ~ 1.10)	1.05 (0.92 ~ 1.20)	< 0.001
Alkaline phosphatase (U/L)	67 (56 ~ 82)	68 (57 ~ 81)	67 (56 ~ 82)	0.998	66 (55 ~ 79)	65 (55 ~ 79)	66 (55 ~ 81)	0.089
Blood urea nitrogen (mg/dL)	14 (11 ~ 17)	13 (11 ~ 16)	16 (13 ~ 20)	< 0.001	14 (11 ~ 17)	13 (11 ~ 16)	16 (13 ~ 20)	< 0.001
C-reactive protein (mg/dL)	0.19 (0.08 ~ 0.40)	0.18 (0.08 ~ 0.38)	0.21 (0.09 ~ 0.43)	< 0.001	0.17 (0.08 ~ 0.36)	0.17 (0.08 ~ 0.35)	0.19 (0.09 ~ 0.41)	< 0.001
lymphocyte percentage (%)	28.30 (23.30 ~ 34.20)	29.90 (24.80 ~ 35.30)	26.10 (20.80 ~ 31.30)	< 0.001	28.40 (23.40 ~ 33.70)	29.20 (24.40 ~ 34.20)	25.60 (20.40 ~ 30.90)	< 0.001
Mean cell volume (fL)	91.10 (88.00 ~ 94.10)	90.30 (87.50 ~ 93.20)	92.20 (89.10 ~ 95.20)	< 0.001	90.90 (88.10 ~ 93.80)	90.57 (87.80 ~ 93.40)	92.30 (89.40 ~ 95.30)	< 0.001
Red cell distribution width (%)	12.60 (12.30 ~ 13.30)	12.50 (12.20 ~ 13.10)	12.90 (12.50 ~ 13.60)	< 0.001	12.50 (12.20 ~ 13)	12.50 (12.10 ~ 12.90)	12.90 (12.40 ~ 13.50)	< 0.001
White blood cell count (1000 cells/uL)	6.80 (5.60 ~ 8.10)	6.90 (5.60 ~ 8.20)	6.80 (5.70 ~ 8.10)	0.452	6.90 (5.60 ~ 8.20)	6.90 (5.60 ~ 8.30)	6.80 (5.70 ~ 8.10)	0.31
Calculated biological age								
KDMAge	46.50 (35.96 ~ 59.45)	40.65 (31.93 ~ 51.11)	57.24 (46.98 ~ 70.13)	< 0.001	41.91 (32.59 ~ 53.22)	38.75 (30.46 ~ 48.43)	54.73 (44.77 ~ 67.22)	< 0.001
KDMAge acceleration	-12.33 (-21.54 ~ -1.96)	-10.51 (-18.68 ~ -1.09)	-16.24 (-26.71 ~ -4.49)	< 0.001	-12.57 (-20.79 ~ -3.30)	-11.39 (-18.96 ~ -2.77)	-17.78 (-27.26 ~ -6.08)	< 0.001
Accelerated KDMAge (n %)	1546 (21.45)	1040 (23.01)	506 (18.82)	< 0.001	9,912,530 (18.33)	7,871,303 (19.05)	2,041,227 (16)	
PhenoAge	56.41 (45.22 ~ 69)	47.72 (41.47 ~ 55.04)	72.39 (66.32 ~ 78.85)	< 0.001	50.02 (42.41 ~ 61.46)	46.27 (40.75 ~ 52.71)	70.78 (64.93 ~ 77.51)	< 0.001
PhenoAge acceleration	-3.22 (-5.93 ~ 0.25)	-3.75 (-6.38 ~ -0.78)	-2.12 (-5.12 ~ 1.98)	< 0.001	-3.73 (-6.27 ~ -0.75)	-4.07 (-6.49 ~ -1.24)	-2.37 (-5.28 ~ 1.36)	< 0.001
Accelerated PhenoAge (n %)	1883 (26.12)	942 (20.84)	941 (34.99)	< 0.001	11,239,728 (20.79)	7,028,734 (17.02)	4,210,994 (33)	
Prostate cancer (n %)	318 (4.41)	47 (1.04)	271 (10.08)	< 0.001	1,548,225 (2.86)	323,890 (0.78)	1,224,335 (9.60)	
Total PSA (ng/mL)	1 (0.60 ~ 1.88)	0.82 (0.52 ~ 1.40)	1.60 (0.80 ~ 3.34)	< 0.001	0.90 (0.54 ~ 1.58)	0.80 (0.50 ~ 1.30)	1.50 (0.80 ~ 3.10)	< 0.001
Free PSA (ng/mL)	0.28 (0.18 ~ 0.48)	0.24 (0.16 ~ 0.37)	0.43 (0.25 ~ 0.79)	< 0.001	0.26 (0.17 ~ 0.42)	0.24 (0.16 ~ 0.36)	0.42 (0.24 ~ 0.76)	< 0.001
PSA ratio (%)	28 (21 ~ 38)	28 (21 ~ 38)	28 (21 ~ 37)	0.123	29 (22 ~ 38)	30 (22 ~ 39)	29 (22 ~ 37)	0.011

*BMI* body mass index; *ALT* alanine aminotransferase; *AST* aspartate aminotransferase; *KDMAge* Klemera-Doubal method age; *PhenoAge* phenotypic age; *PSA* prostate-specific antigen; KDMAge acceleration, the residual of the regression of KDMAge based on chronological age; PhenoAge acceleration, the residual of the regression of PhenoAge based on chronological age; Accelerated KDMAge, KDMAge acceleration greater than 0; Accelerated PhenoAge, PhenoAge acceleration less than 0

### Association of biological age measures with risk of PCa

**Fig. 1:**
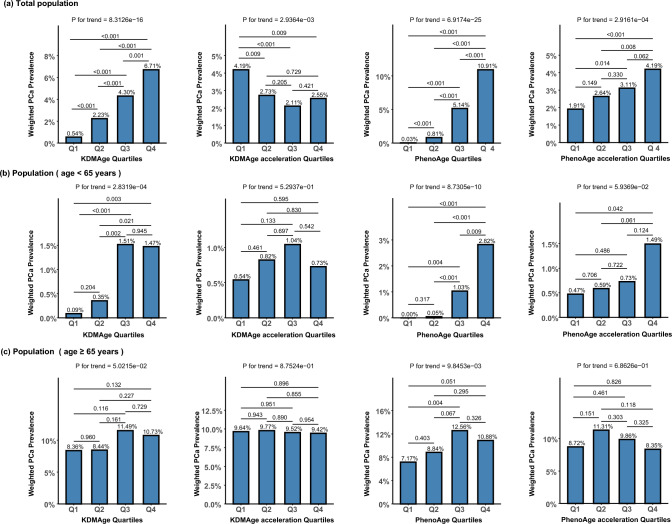
Association of Biological Age Measures with Risk of PCa. (**a**) Total population. (**b**) Population (age < 65 years). (**c**) Population (age ≥ 65 years). KDMAge, Klemera-Doubal method age; KDMAge acceleration, the residual of the regression of KDMAge based on chronological age; PhenoAge, phenotypic age; PhenoAge acceleration, the residual of the regression of PhenoAge based on chronological age; *Pca* prostate cancer; *Q1* quartile 1; *Q2* quartile 2; *Q3* quartile 3; *Q4* quartile 4

When the population analyzed for PCa was stratified into quartiles based on various biological age measures, a noticeable increasing trend in the prevalence of PCa with rising quartiles of both KDMAge and PhenoAge was observed in the overall population (P for trend < 0.001). Furthermore, there was a significant increasing trend in the prevalence of PCa with rising quartiles of PhenoAge acceleration (P for trend < 0.05), though this trend was not observed across KDMAge acceleration quartiles (Fig. 1a). After stratification by age, while the prevalence of PCa was generally lower in the younger group compared to the older group, increasing trends across quartiles of KDMAge, PhenoAge, and PhenoAge acceleration were observed, whereas similar trends disappeared in the older group (Figs. 1b and 1c). After adjustment for potential confounding factors including age, BMI, race, diabetes history, hypertension history, smoking status, and alcohol consumption, an increase of one standard deviation in PhenoAge levels was associated with an approximately two-fold increase in the risk of PCa (OR = 2.08, 95% CI 1.16–3.74, P = 0.015) in the younger group. Similarly, in the younger group, an increase of one standard deviation in PhenoAge acceleration was associated with approximately a 50% increase in the risk of PCa (OR = 1.50, 95% CI 1.09–2.07, P = 0.015), and the accelerated PhenoAge group had a more than three-fold increase in prostate cancer risk compared to the non-accelerated PhenoAge group (OR = 3.65, 95% CI 1.39–9.55, P = 0.009). However, no significant associations between any biological aging measures and the risk of PCa were observed in the older group (Table 2).

**Table 2:** Association between biological age measures and risk of prostate cancer

		Model 1		Model 2		Model 3	
		OR (95% CI)	P value	OR (95% CI)	P value	OR (95% CI)	P value
Age < 65 years							
	KDMAge	1.21 (0.86–1.71)	0.263	1.30 (0.91–1.86)	0.148	1.37 (0.95–1.97)	0.088
	KDMAge acceleration	1.20 (0.87–1.65)	0.263	1.28 (0.91–1.79)	0.148	1.34 (0.96–1.88)	0.088
	Accelerated KDMAge	1.13 (0.35–3.71)	0.833	1.26 (0.38–4.17)	0.702	1.34 (0.41–4.39)	0.626
	PhenoAge	1.53 (0.85–2.76)	0.156	1.79 (0.94–3.39)	0.074	2.08 (1.16–3.74)	0.015
	PhenoAge acceleration	1.26 (0.91–1.75)	0.156	1.38 (0.97–1.96)	0.074	1.50 (1.09–2.07)	0.015
	Accelerated PhenoAge	2.24 (0.93–5.38)	0.070	2.72 (1.07–6.91)	0.035	3.65 (1.39–9.55)	0.009
Age ≥ 65 years							
	KDMAge	0.95 (0.82–1.09)	0.445	0.97 (0.84–1.13)	0.723	0.99 (0.85–1.16)	0.883
	KDMAge acceleration	0.95 (0.83–1.09)	0.445	0.98 (0.85–1.12)	0.723	0.99 (0.85–1.15)	0.883
	Accelerated KDMAge	0.91 (0.61–1.37)	0.648	0.97 (0.65–1.45)	0.879	1.00 (0.66–1.51)	0.995
	PhenoAge	0.85 (0.68–1.06)	0.140	0.88 (0.71–1.08)	0.210	0.90 (0.72–1.11)	0.317
	PhenoAge acceleration	0.89 (0.76–1.04)	0.140	0.91 (0.79–1.06)	0.210	0.93 (0.79–1.08)	0.317
	Accelerated PhenoAge	0.69 (0.49–0.98)	0.037	0.72 (0.52–1.01)	0.056	0.74 (0.52–1.06)	0.098

Model 1: Adjusted for age and race; Model 2: Additionally adjusted for obesity, history of hypertension, and history of diabetes, based on Model 1; Model 3: Further adjusted for smoking status and alcohol consumption, based on Model 2. Results are expressed as odds ratios (OR) with 95% confidence intervals (CI) and P-values

### Association of biological age measures with risk of highly-probable PCa

**Fig. 2:**
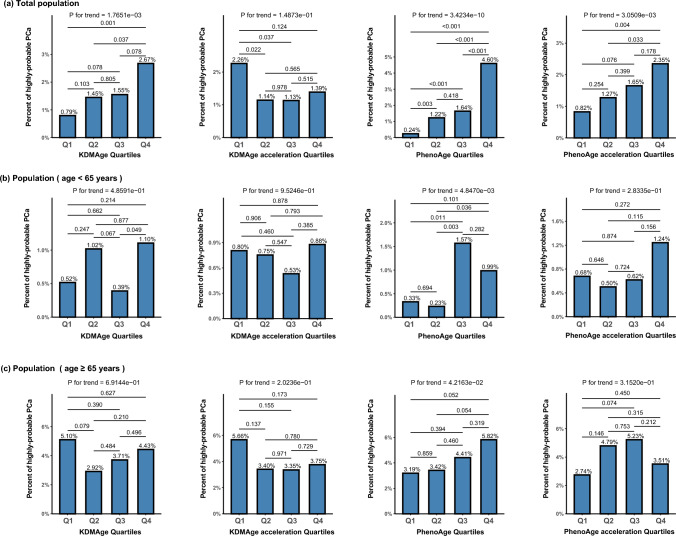
Association of Biological Age Measures with Risk of Highly-Probable PCa. (**a**) Total population. (**b**) Population ( age < 65 years). (**c**) Population (age ≥ 65 years). *KDMAge* Klemera-Doubal method age; *KDMAge acceleration* the residual of the regression of KDMAge based on chronological age; *PhenoAge* phenotypic age; *PhenoAge acceleration* the residual of the regression of PhenoAge based on chronological age; *Pca* prostate cancer; *Q1* quartile 1; *Q2* quartile 2; *Q3* quartile 3; *Q4* quartile 4

Highly-probable PCa was defined by combining multiple PSA indicator levels (as detailed in the Methods section). When the population analyzed for PSA was stratified into quartiles based on various biological age measures, similar to PCa, a noticeable increasing trend in the percentage of highly-probable PCa with rising quartiles of both KDMAge and PhenoAge levels was observed in the overall population (P for trend < 0.05). Furthermore, there was a significant increasing trend in the percentage of highly-probable PCa with rising quartiles of PhenoAge acceleration (P for trend < 0.05), though this trend was not observed across KDMAge acceleration quartiles (Fig. 2a). After stratification by age, only in the older group was an increasing trend observed across quartiles of PhenoAge (P for trend < 0.05) (Fig. 2c). After adjustment for potential confounding factors, including age, BMI, race, diabetes history, hypertension history, smoking status, and alcohol consumption, an increase of one standard deviation in PhenoAge levels was associated with approximately a 56% increase in the risk of highly-probable PCa (OR = 1.56, 95% CI 1.04–2.34, P = 0.031) in the younger group. Similarly, in the younger group, an increase of one standard deviation in PhenoAge acceleration was associated with approximately a 28% increase in the risk of highly-probable PCa (OR = 1.28, 95% CI 1.02–1.60, P = 0.031) (Table 3).

**Table 3:** Association between biological age measures and risk of highly-probable prostate cancer

		Model 1		Model 2		Model 3	
		OR (95% CI)	P value	OR (95% CI)	P value	OR (95% CI)	P value
Age < 65 years							
	KDMAge	1.03 (0.66–1.61)	0.885	1.05 (0.63–1.74)	0.851	1.03 (0.62–1.70)	0.922
	KDMAge acceleration	1.03 (0.68–1.56)	0.885	1.05 (0.65–1.68)	0.851	1.02 (0.64–1.65)	0.922
	Accelerated KDMAge	1.40 (0.51–3.88)	0.510	1.48 (0.41–5.34)	0.540	1.41 (0.39–5.09)	0.599
	PhenoAge	1.61 (1.02–2.56)	0.043	1.67 (1.07–2.61)	0.026	1.56 (1.04–2.34)	0.031
	PhenoAge acceleration	1.30 (1.01–1.68)	0.043	1.33 (1.04–1.70)	0.026	1.28 (1.02–1.60)	0.031
	Accelerated PhenoAge	1.96 (0.77–4.97)	0.156	2.02 (0.80–5.10)	0.136	1.84 (0.78–4.33)	0.161
Age ≥ 65 years							
	KDMAge	0.89 (0.66–1.20)	0.458	0.95 (0.69–1.29)	0.719	0.94 (0.70–1.28)	0.704
	KDMAge acceleration	0.90 (0.67–1.20)	0.458	0.95 (0.70–1.28)	0.719	0.95 (0.71–1.26)	0.704
	Accelerated KDMAge	1.01 (0.58–1.77)	0.968	1.15 (0.65–2.05)	0.631	1.15 (0.66–2.01)	0.614
	PhenoAge	0.99 (0.76–1.29)	0.941	1.11 (0.86–1.44)	0.431	1.11 (0.87–1.43)	0.404
	PhenoAge acceleration	0.99 (0.82–1.20)	0.941	1.08 (0.90–1.29)	0.431	1.08 (0.90–1.29)	0.404
	Accelerated PhenoAge	1.02 (0.70–1.47)	0.932	1.21 (0.84–1.75)	0.295	1.22 (0.85–1.74)	0.273

Model 1: Adjusted for age and race; Model 2: Additionally adjusted for obesity, history of hypertension, and history of diabetes, based on Model 1; Model 3: Further adjusted for smoking status and alcohol consumption, based on Model 2. Results are expressed as odds ratios (OR) with 95% confidence intervals (CI) and P-values

## Discussion

Our study utilized nationally representative data from the NHANES survey to explore the relationships between two measures of biological age—KDMAge and PhenoAge—and their acceleration adjusted for chronological age, with the risk of PCa and highly-probable PCa as defined by integrated PSA levels. After accounting for confounding factors influencing the risk of PCa, we found that increased PhenoAge acceleration was associated with a higher risk of PCa in individuals younger than 65 years old. Notably, even among those under 65 years old without PCa, elevated PhenoAge acceleration was significantly associated with an increased risk of highly-probable PCa, indicating that biological aging acceleration signals an early increased risk of PCa. These findings provide new insights into the role of biological aging in cancer development and offer novel methods for identifying individuals at early risk of PCa during the aging process.

The aging population and extended life expectancy are leading to an increase in older men, a demographic at higher irreversible risk for various cancers [14, 15]. The development of PCa is also closely linked with increasing age [2, 16–18], with studies showing that newly diagnosed PCa in older individuals has more than tripled from 1990 to 2013 [19]. While prostate cancer diagnoses and related mortalities are rare in males younger than 50 years old, approximately 85% of PCa are diagnosed after the age of 65 years old [2]. Men older than 65 have a higher likelihood of adverse pathological findings at radical prostatectomy compared to their younger counterparts (adjusted OR, 1.28; 95% CI 1.00–1.62; P = 0.048) [20]. The mean age of patients with PCa ranges from 72–74 years, with most deaths occurring in men older than 75 years [2, 21]. Autopsy studies suggest that most men over 85 years old have histological PCa [2]. A Swedish population-based PCa screening trial demonstrated that age is linked with the risk of clinically significant PCa, with each one-year increase in age increasing the risk of being diagnosed with a higher Gleason score, indicative of more aggressive PCa [22]. Data from the population-based SEER database show that the 10-year risk of PCa increases from 2.3% at age 50 to 7.3% at age 70, with about 60% of diagnosed cases being men over 65 years old [16]. Furthermore, analysis of large prostate biopsy datasets reveals that older men have larger prostate volumes and increased risks of abnormal DRE and higher Gleason scores[16].

However, the clinical pattern relating chronological age with PCa has also changed noticeably over the past few years [23]. The proportion of men diagnosed before the age of 70 and the proportion of moderately differentiated tumors have both increased. The age at death from PCa peaks between 80–84 years old [24]. A study assessing trends in metastatic PCA in the American population showed that the annual growth rate of metastatic PCA in men under 69 was faster than in older men, with the highest projected growth rates in men aged 45–54 [4]. Moreover, national data from the SEER Program revealed that more men were diagnosed with PCa at a younger age and earlier stage in 2004–2005 compared to previous years, with the average age at diagnosis decreasing from 72.2 to 67.2 years [25]. These evidences suggest that chronological age alone cannot fully explain the increasing incidence of PCA in younger men, highlighting the limitations of chronological age in explaining the onset of PCa related to aging. The real physiological and pathological disorders underlying age progression are better explained by factors independent of chronological age, providing a more accurate identification of the residual risk of PCa.

Our study employed a set of common clinical physical examination and blood test markers to calculate two biological age indicators, KDMAge and PhenoAge, and their accelerated versions adjusted for actual age, better reflecting the degree of accelerated aging in individuals of the same age. By analyzing the correlation between biological age acceleration and the risk of PCa as well as early highly-probable PCa defined by PSA levels, we found significant associations in younger individuals under 65 years old, but not in older adults. In contemporary society, the increasing prevalence of metabolic abnormalities such as hypertension, diabetes, and obesity among younger people, coupled with more young individuals experiencing excessive mental stress and adopting poor lifestyle habits like staying up late, smoking, and excessive drinking, makes younger individuals more susceptible to biological aging. This also explains the increasing trend of younger individuals being diagnosed with PCa. More importantly, we found that in younger individuals without PCa, elevated biological aging acceleration was significantly associated with an increased early risk of PCa as defined by PSA levels, which can identify treatable early-stage PCa, significantly reducing disease-specific mortality and improving the detection of asymptomatic, well-differentiated PCa [26, 27].

Our results are strengthened by a nationally representative sample, which applied survey weights to ensure the generalizability of our findings to adult men in the USA, as well as obtaining comprehensive data on numerous essential covariates through the integration of NHANES data. Moreover, the strong correlation between chronological age and PCa was thoroughly considered by adjusting for age during the calculation of biological age acceleration, and a relatively detailed analysis was conducted in different age subgroups. Nevertheless, our study has some limitations. First, as a cross-sectional analysis, it inherently limits the ability to establish a definitive causal relationship. Second, we utilized self-reported physician-diagnosed cases of PCa, which may introduce recall bias due to the limitations of self-reported methods. Third, histories of diabetes and hypertension were identified through self-reported diagnoses instead of direct blood pressure or glucose measurements, which may lead to the omission of currently affected individuals and potential recall bias. Additionally, the lack of data on tumor stage and grade means we could not account for the severity of cancers. Finally, due to the limited number of study participants, we were unable to fully consider the relationship between biological age and PCa across different races. Therefore, caution is advised during the analysis and interpretation of the data. In the future, prospective studies involving tumor grading and different racial subgroups are essential to provide stronger evidence for our findings.

## Conclusion

In conclusion, our findings provide new insights into the increasingly younger age of onset of PCa, linking accelerated biological aging with higher risks of PCa in younger men. Importantly, our study offers novel methods for identifying individuals at early risk of PCa during the aging process.

## Supplementary Information

Below is the link to the electronic supplementary material.

Supplementary file1 (PDF 142 KB)

## Data Availability

All NHANES datasets used in this study are publicly available and listed by survey cycle at wwwn.cdc.gov/nchs/nhanes.
