# Supplementary figures and images for: Association of biological aging with prostate cancer: insights from the National Health and Nutrition Examination Survey
Source: Aging Clin Exp Res. 2024 Oct 24;36(1):209. doi: 10.1007/s40520-024-02861-0 (PMC11502538; doi:10.1007/s40520-024-02861-0)

**Figure S1**

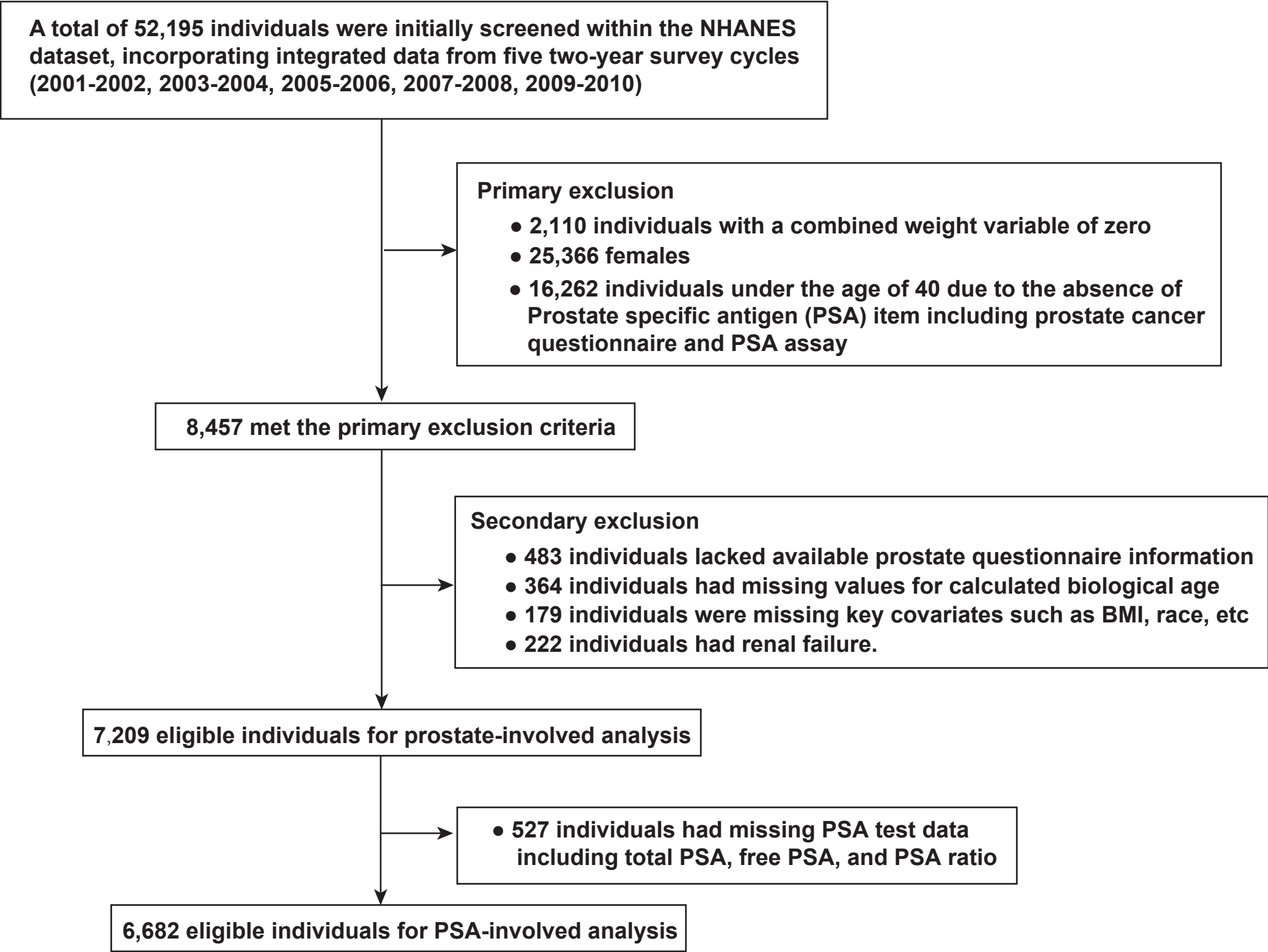

Supplement: Supplementary file 1 — Supplementary file1 (PDF 142 KB) [file 40520_2024_2861_MOESM1_ESM.pdf]
